# Use of lecanemab for the treatment of Alzheimer's disease: A systematic review

**DOI:** 10.1002/brb3.3592

**Published:** 2024-06-12

**Authors:** Md Fahad Hossain, Ashma Ul Husna, Manish Kharel

**Affiliations:** ^1^ Ministry of Health and Family Welfare Dhaka Bangladesh; ^2^ Mercy Health St. Elizabeth Youngstown Hospital Youngstown Ohio USA; ^3^ Department of Medicine Kathmandu Medical College Kathmandu Nepal

**Keywords:** Alzheimer's, lecanemab, Leqembi

## Abstract

**Purpose:**

The US Food and Drug Administration authorized lecanemab for the therapeutic use of Alzheimer's disease (AD) in January 2023. To assess the effectiveness and safety of lecanemab in treating AD, we thoroughly examined the studies that are currently accessible.

**Method:**

Preferred Reporting Items for Systematic Reviews and Meta‐Analysis recommendations were followed. In order to find relevant studies on lecanemab, we carried out a thorough literature search utilizing the electronic databases MEDLINE via PubMed, Cochrane, Web of Science, EBSCOhost, and Scopus. Excluding any research using experimental animals, we looked at lecanemab's effectiveness and side effects in treating AD in human clinical trials. Three randomized controlled studies were included.

**Findings:**

According to studies, lecanemab lessens clinical deterioration and reduces brain amyloid‐beta plaques (difference,.45; 95% confidence interval,.67 to.23; *p* < .001). Participants who received lecanemab saw a greater frequency of amyloid‐related imaging abnormalities (ARIA)‐H (17.3% vs. 9.0%) and ARIA‐E (12.6% vs. 1.7%), which is a significant adverse outcome.

**Conclusion:**

Lecanemab has been shown to have an impact on the two primary pathophysiologic indicators of AD (Aβ and tau). There are still a lot of unresolved issues related to lecanemab. Future research on the effectiveness and safety of lecanemab is advised in order to determine that the advantages of this medication exceed the disadvantages.

## Graphical abstract

Immunotherapy has been showing promising results in the treatment of Alzheimer's disease. Lecanemab is one of the crucial immunomodulating drugs. But further study is required to learn more.

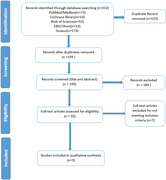

## 1 INTRODUCTION

Alzheimer's disease (AD), also known as dementia, is a serious neurological condition marked by behavioral abnormalities, memory loss, and progressive cognitive deterioration. Although the exact causes of AD are still unknown, it is commonly accepted that the buildup of amyloid beta (Aβ) oligomers plays a significant role in the disease's etiology (Goedert & Spillantini, 2006; Klein et al., 2001). Immunotherapies targeting those oligomers have been showing amazing results in treating AD (Klein et al., 2001; Wisniewski & Goñi, 2015). Lecanemab, also known as BAN2401, is a monoclonal antibody that has been developed to treat AD (Swanson et al., 2018). It selectively targets Aβ oligomers. There is hope for a potential new treatment for AD after this medicine demonstrated excellent results in preclinical research and Phases 2 and 3 clinical trials (Golde et al., 2011).

The buildup of intracellular neurofibrillary tangles and extracellular amyloid plaques in the brain, which are thought to impair neuronal function and lead to cognitive decline (Goedert & Spillantini, 2006; Haass & Selkoe, 2007; Sperling et al., 2011), is the pathological characteristic of AD. The amyloid precursor protein (APP) is broken down into the peptide A, which can then assemble into harmful oligomers for neurons (Jack & Holtzman, 2013; Karran et al., 2011). The cognitive and behavioral abnormalities associated with AD are considered to be caused by A oligomers, which disrupt synapse function and encourage neuronal cell death (Mucke & Selkoe, 2012; Sperling et al., 2011).

A monoclonal antibody called lecanemab preferentially binds to A oligomers to stop them from aggregating into amyloid plaques (Cummings et al., 2019; Eisai & Biogen, 2021; Logovinsky et al., 2016; Sevigny et al., 2016). Lecanemab has been demonstrated in preclinical investigations to decrease A oligomer levels in the brain and to enhance cognitive performance in animal models of AD (Logovinsky et al., 2016; Sevigny et al., 2016). These encouraging findings prompted the creation of clinical trials to examine the security and effectiveness of lecanemab in people.

Lecanemab was reported to lower A levels in the brain in a dose‐dependent manner and to halt cognitive loss in individuals with early AD in a Phase 2 clinical trial (Swanson et al., 2018). Based on these findings, a Phase 3 clinical trial with more than 1500 individuals with early AD was started. The trial's primary goal, which demonstrated a statistically significant decrease in cognitive deterioration in individuals who got the highest dose of lecanemab compared to placebo, was reached in June 2021, according to an announcement from Eisai and Biogen (2021).

## 2 METHODS

We followed the PRISMA (Preferred Reporting Items for Systematic Reviews and Meta‐Analysis) standards for conducting this systematic review (Page et al., 2021). The protocol is registered in PROSPERO (CRD42023422725).

### 2.1 Data sources

We have searched various databases, which include MEDLINE through PubMed, Web of Science, The Cochrane Library (Cochrane Central Register of Controlled Trials‐CENTRAL), EBSCOhost, and Scopus with a search strategy using the keywords Lecanemab, Leqembi, “Lecanemab‐irmb,” Alzheimer, Alzheimer's, “Alzheimer's disease,” “Alzheimer Disease,” “APP,” “Amyloid beta‐Peptides,” “amyloid plaque,” “Temporoparietal atrophy,” “Neurodegenerative disease,” “Neurofibrillary tangles.” The search was done on March 31, 2023.

### 2.2 Study selection

We searched for articles considering the benefits and adverse effects of lecanemab in the treatment of AD. We considered randomized controlled trials (RCTs), quasi‐experimental studies, non‐randomized trials, controlled before‐after studies, analytic studies (cohort study and case–control study), and comparative cross‐sectional studies that are published in English only.

We have excluded the study not considering lecanemab, studies not focusing on AD, laboratory experiments, incomplete or ongoing studies, animal studies, reviews, case series, case reports, letters, comments, editorials, book chapters, and opinions.

The title and abstract of the redeemed articles were reviewed individually by two reviewers, and any disagreements were resolved by a third reviewer. To screen the articles, we used the internet tool Rayyan (Ouzzani et al., 2016). Then, two independent teams went through the full‐text articles, and the lead reviewer solved any disagreement. We excluded the articles following the “prioritization and sequential exclusion” technique (Saif‐Ur‐Rahman et al., 2022). Reasons for exclusion were reported.

### 2.3 Data extraction

We gathered information based on the study population, the duration and dosage of the intervention, the positive and negative results, the gravity of the bad results, and other factors. Two reviewers extracted data independently, and the lead reviewer cross‐checked to resolve any dispute.

### 2.4 Data analysis

We have performed a narrative synthesis here. Due to the variation of findings, a meta‐analysis could not be done.

### 2.5 Risk of bias assessment

The risk of bias (ROB) was evaluated using the Cochrane ROB assessment method. The ROB was evaluated independently by two teams of review authors, and any disagreements were settled by discussion. Random sequence generation, participant and personnel blinding, allocation concealment, blinding of outcome assessors, inadequate outcome data, selective reporting, and other biases were the domains taken into consideration in ROB.

## 3 RESULTS

### 3.1 Search results

**FIGURE 1:**
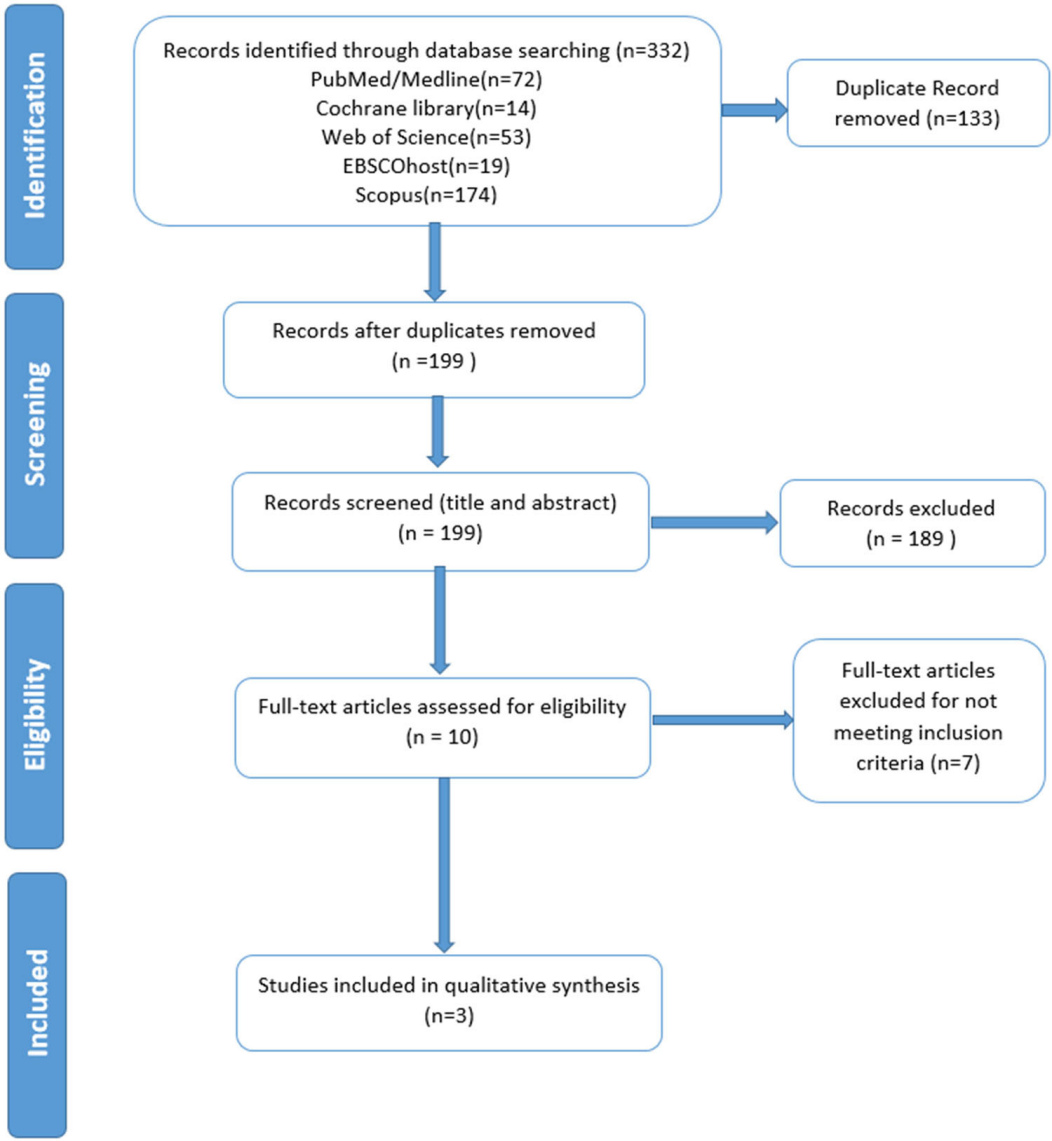
Preferred Reporting Items for Systematic Reviews and Meta‐Analysis (PRISMA) flow diagram.

The comprehensive search from five databases retrieved 332 articles. After removing 133 duplicates, we listed a total of 199 articles for screening the title and abstract. At this stage, 189 articles were excluded due to not meeting inclusion criteria as mentioned in Section 2. Among the remaining articles, seven were excluded in the phase of full‐text screening. Due to their lack of relevance to the research intervention, these articles were eliminated. Three RCTs were ultimately included in the analysis. PRISMA flow diagram of the detailed selection process of included articles is represented in Figure 1

### 3.2 ROB assessments of clinical trials

**FIGURE 2:**
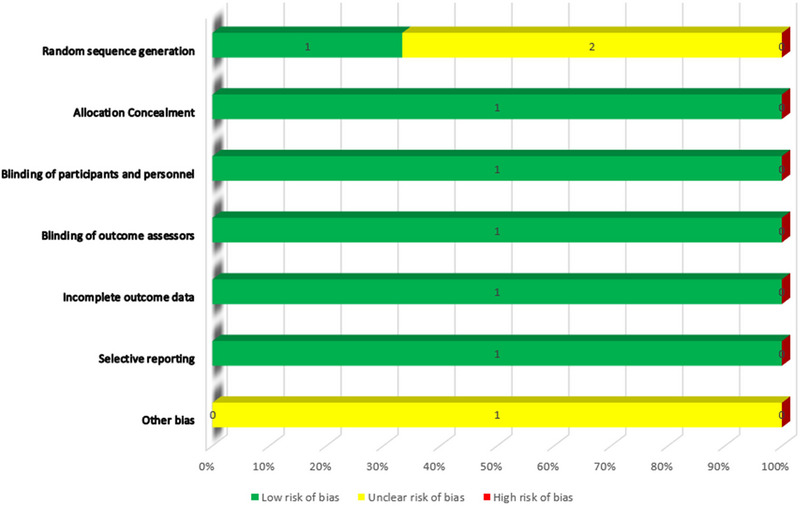
Cochrane risk of bias (ROB) assessment diagram.

We used the ROB tool to assess the ROB. One out of three gave information about random sequence generation. Allocation concealment was done for all the articles. All the participants and personnel were also blinded about the outcome accessor of data so we rated them as low ROB. No articles had incomplete outcome data or there was no selective reporting done. So the ROB was also low. But no study mentions anything about any other ROB. As there was no information about any other bias risk so we put all articles under unclear risk for other biases. Figure 2 shows the graphical presentation of ROB.

The listed research was all released between 2016 and 2023. They were all RCTs. Males and females were both present in every trial. The trials included subjects who were older than 50. Lecanemab dosages were compared to a placebo in three studies to compare their effects. One study's intervention lasted for 18 months, another for 12 months, and one more for 4 months. Two studies examined both positive and negative outcomes, whilst the other exclusively examined the negative impacts of the intervention. Table 1 lists the characteristics of the publications that were included as well as a summary of the results.

**TABLE 1:** Characteristics of included studies.

Authors	Year of publication	Sample size	Study design	Country	Age in years	Gender	Intervention	Duration of intervention	Outcome
vanDyck et al.	2023	Control: 859 Intervention:875	RCT PHASE 3		50–90 years	Male and female	Lecanemab (10 mg/kg) vs. placebo	18	Primary end point:10 mg/kg lecanemab declined CDR‐SB at 18 months: adjusted mean difference vs. placebo (95% CI) −.45 (−.67 to −.23); *p* < .001
Swanson et al.	2021	Control:245 Intervention: 609	RCT phase 2 b	North America (the USA and Canada), Europe (France, Germany, Italy, the Netherlands, Spain, Sweden, and the UK), and the Asia‐Pacific region (Japan and South Korea)	≥50 years	Male and female	Lecanemab (2.5 mg/kg biweekly, 5 mg/kg monthly, 5 mg/kg bi‐weekly, 10 mg/kg monthly, 10 mg/kg biweekly) vs. placebo	Primary end point at 12 month Secondary end point at 18 month	Primary end point: the 10‐mg/kg biweekly ED90 dose showed a 64% probability to be better than placebo by 25% on ADCOMS, which missed the 80% threshold for the primary outcome Secondary end point:10 mg/kg biweekly on ADCOMS (27%; with 97.7% probability to be superior to placebo), CDR‐SB (33%; with 96.4% probability to be superior to placebo), and ADAS‐Cog14 (56%, with 98.8% probability to be superior to placebo
Logovinsky et al.	2016	SAD cohort placebo:12 Intervention: 36 0.1:6, 0.3:6, 1:6, 3:6, 10:6, 15:6 MAD cohort placebo:8 Intervention: 24 0.3:6, 1:6, 3:6, 10:6	RCT phase 2b study		≥50 years	Male and female	Single ascending dose (SAD) study lecanema. doses of 0.1, 0.3, 1, 3, 10, and 15 mg/kg. A multiple ascending dose (MAD) lecanemab doses of 0.3, 1, 3 mg/kg every 4 week and 10 mg/kg biweekly	4 months	Incidence. of ARIA‐E/H on MRI was comparable to that of placebo

Abbreviations: ARIA, amyloid‐related imaging abnormalities; CDR‐SB, Clinical Dementia Rating Scale Sum Boxes; CL, confidence interval.

### 3.3 Description of intervention

Van Dyck et al. (2023) measured outcome of lecanemab 10 mg per/kg or placebo every 2 weeks for 18 months. Swanson et al. (2018) investigated the five intravenous lecanemab dosages (2.5 mg/kg biweekly, 5 mg/kg monthly, 5 mg/kg biweekly, 10 mg/kg monthly, 10 mg/kg biweekly, or placebo) across 12 months for the primary outcome and 18 months for the secondary endpoint. Lecanemab or placebo was studied intravenously at dosages of 0.1, 0.3, 1, 3, 10, and 15 mg/kg for single ascending doses and at doses of 0.3, 1, 3 mg/kg every 4 weeks and 10 mg/kg biweekly for multiple ascending doses (MAD) administered over the course of seven treatments over a period of 4 months by Logovinsky et al. (2016). Table 2 demonstrates the in detail outcome of intervention with doses and summary.

**TABLE 2:** Description of outcomes.

Authors	Intervention	Effectiveness measurement		Adverse effects	
		Intervention	Control	Intervention	Control
vanDyck et al.	Lecanemab (10 mg/kg) vs. placebo	Primary end point (mean change) CDR‐SB: 1.21 Secondary end point (mean change) amyloid burden on PET: −55.48 centiloids ADAS‐cog14 score: 4.14 ADCOMS: 0.164 ADCS‐ADL‐MCI: −3.5	Primary endpoint (mean change) CDR‐SB: 1.66 Secondary end point (mean change) amyloid burden on PET: 3.64 centiloids ADAS‐cog14 score: 5.58 ADCOMS: 0.214 ADCS‐ADL‐MCI: −5.5	Any adverse event 798(88.9) Adverse event lead to discontinuation of trial 62(6.9) Infusion‐related reaction 237 (26.4) Fall 93(10.4) Dizziness 49(5.5) Anxiety 45(5.0) Diarrhea: 48(5.3) UTI 78 (8.7). ARIA‐E: 113 (12.6) ARIA‐H: microhemorrhage 126 (14.0)	Any adverse event 735(81.9) Adverse event lead to discontinuation of trial 26(2.9) Infusion‐related reaction 66 (7.4) Fall 86(9.6) Dizziness 46(5.5) Anxiety 38(4.2) Diarrhea: 58(6.5) UTI 82 (9.1 ARIA‐E: 15 (1.7 ARIA‐H: microhemorrhage 68 (7.6)
Swanson et al.	Lecanemab (2.5 mg/kg biweekly, 5 mg/kg monthly, 5 mg/kg bi‐weekly, 10 mg/kg monthly, 10 mg/kg biweekly) vs. placebo	Primary end point 2.5 mg/kg biweekly: 0.134 ± 0.024 5 mg/kg monthly: 0.119 ± 0.021 5 mg/kg biweekly: 0.116 ± 0.016 10 mg/kg monthly: 0.084 ± 0.011 10 mg/kg biweekly: 0.077 ± 0.014 Secondary end point amyloid PET SUVr (least squares mean changes) 2.5 mg/kg biweekly: −0.094 5 mg/kg monthly: −0.131 5 mg/kg biweekly: −0.197 10 mg/kg monthly: −0.225, 10 mg/kg biweekly: −0.306 ADCOMs: 10 mg/kg monthly:146/246 10 mg/kg biweekly: 79/152 CDR‐SB: 10 mg/kg monthly: 149/246 10 mg/kg biweekly: 84/152 ADAS‐cog: 10 mg/kg monthly:146/246 10 mg/kg biweekly: 79/152	Primary end point Placebo: 0.113 ± 0.012 secondary end point amyloid PET SUVr (Least squares mean changes) Placebo: 0.004 ADCOMs: placebo:160/238 CDR‐SB: placebo: 161/238 ADAS‐cog: placebo: 158/238	Any TEAE 2.5 mg/kg biweekly: 88.5% 5 mg/kg monthly: 94.1% 5 mg/kg biweekly: 88% 10 mg/kg monthly: 94.1% 10 mg/kg biweekly: 86.3% serious adverse event 2.5 mg/kg biweekly: 19.2% 5 mg/kg monthly: 7.8% 5 mg/kg biweekly: 17.4% 10 mg/kg monthly: 12.3% 10 mg/kg biweekly:15.5% leading to discontinuation 2.5 mg/kg biweekly: 13.5% 5 mg/kg monthly: 7.8% 5 mg/kg biweekly: 10.9% 10 mg/kg monthly: 18.6% 10 mg/kg biweekly: 14.9% ARIAE 2.5 mg/kg biweekly: 1.9% 5 mg/kg monthly: 2% 5 mg/kg biweekly: 3.3% 10 mg/kg monthly: 9.9% 10 mg/kg biweekly: 9.9%	Any TEAE Placebo: 88.2% serious adverse event Placebo: 17.6% leading to discontinueation Placebo: 6.1% ARIAE Placebo: 0.8%
Logovinsky et al.	Single ascending dose (SAD) study lecanemab. doses of 0.1, 0.3, 1, 3, 10, and 15 mg/kg. A multiple ascending doses (MAD) lecanemab doses of 0.3, 1, 3 mg/kg every 4 weeks and 10 mg/kg biweekly			SAD cohort Any TEAE 0.1 mg/kg: 6(100%) O.3 mg/kg: 1(16.7%) 1 mg/kg: 3(50%) 3 mg/kg: 3(50%) 10 mg/kg: 2(33.3%) 15 mg/kg: 5(83.3%) Dizziness 0.1 mg/kg: 2(33.3%) O.3 mg/kg: 0 1 mg/kg: 1(16.7%) 3 mg/kg: 0 10 mg/kg: 0 15 mg/kg: 0 Fatigue 0.1 mg/kg: 0 O.3 mg/kg: 0 1 mg/kg: 0 3 mg/kg: 0 10 mg/kg: 0 15 mg/kg: 2(33.3%) Sinusitis 0.1 mg/kg: 1(16.7) O.3 mg/kg: 0 1 mg/kg: 0 3 mg/kg: 1(16.7) 10 mg/kg: 0 15 mg/kg: 2(33.3%) MAD cohort Any TEAE 0.1 mg/kg: 4(66.7%) 1 mg/kg: 3(50%) 3 mg/kg: 4(66.7%) 10 mg/kg: 4(66.7%)	Any TEAE placebo: 8(66.7%) Dizziness placebo: 1(8.3%) Fatigue placebo: 1(8.3%) Sinusitis placebo: 0 MAD cohort Any TEAE placebo:6(75%) Headache placebo: 2(25%) Upper respiratory tract infection placebo: 1(12.5%)
				Headache 0.1 mg/kg: 1(16.7%) 1 mg/kg: 0 3 mg/kg: 1(16.7%) 10 mg/kg: 1(16.7%) Upper respiratory tract infection 0.1 mg/kg: 2(33.3%) 1 mg/kg: 0 3 mg/kg: 0 10 mg/kg: 2(33.3%)	

Abbreviations: ARIA, amyloid‐related imaging abnormalities; CDR‐SB, Clinical Dementia Rating Scale Sum Boxes; TEAEs, treatment‐emergent adverse events.

### 3.4 Effectivenesss

C.H. vanDyck (Ouzzani et al., 2016) and his team evaluated the positive outcomes of lecanemab in an 18‐month trial. The primary endpoint, the clinical decline, was observed in CDR‐SB (Clinical Dementia Rating Scale Sum Boxes) in lecanemab (difference, −.45; 95% confidence interval, −.67 to −.23; *p* < .001). For the secondary endpoint, the clinical decline in the amyloid burden on PET, ADAS‐cog14 score, ADCOMS, and ADCS‐ADL‐MCI: differences −59.12 centroids, −1.44, −0.050, 2 respectively. Swanson et al. (2018) and his team evaluated the positive outcomes of lecanemab in a 12‐month trial. The primary endpoint at 12 months showed a 64% probability to be better than the placebo by 25% on ADCOMS, which missed the 80% threshold for the primary outcome. The secondary endpoint over 10 mg/kg biweekly on ADCOMS (27%; with 97.7% probability to be superior to placebo), CDR‐SB (33%; with 96.4% probability to be superior to placebo), and ADAS‐Cog14 (56%, with 98.8% probability to be superior to placebo).

### 3.5 Safety

C.H. vanDyck (Ouzzani et al., 2016) stated that infusion‐related reactions are the most common adverse event that occurs after a single dose (26.4% with lecanemab vs. 7.4% with placebo). The incidence of amyloid‐related imaging abnormalities (ARIA)‐H (17.3% vs. 9.0%) and ARIA‐E (12.6% vs. 1.7%) was higher in participants who received lecanemab compared to those on placebo. Moreover, the frequency of ARIA‐E and ARIA‐H was numerically larger in ApoE 4 homozygotes than in ApoE 4 heterozygotes, and it was numerically less prevalent in ApoE 4 carriers.

Swanson et al. (2018) investigated most common adverse events that occurred during treatment, which were infusion reactions, with the highest incidence observed in the 10 mg/kg monthly group (22.9%). ARIA‐E was observed in a small percentage of participants 9.9% in 10 mg/kg monthly and 10 mg/kg biweekly dosing regimen. The incidence of ARIA‐E was higher in those who received lecanemab (48 cases) compared to placebo, and a majority of cases occurred in participants who had the ApoE4 gene (37 cases). Of the total cases of ARIA‐E, 5 cases were symptomatic (11% of cases in the lecanemab group). ARIA‐H was observed with a higher incidence in the lecanemab group (10.7). However, there were no clear trends in the incidence of ARIA‐H based on the dosing of lecanemab.

In the study of Logovinsky et al. (2016), dizziness (8.3%), fatigue (5.6%), and sinusitis (5.6%) were the most commonly observed treatment‐emergent adverse events (TEAEs) with a single dose of BAN2401. In subjects who received multiple doses of BAN2401, the most frequently observed TEAEs were upper respiratory tract infection (16.7%), headache (12.5%), and orthostatic hypotension (12.5%). Throughout the entire study, no symptomatic ARIA‐Es, ARIA‐Es, or superficial hemosiderosis were observed in participants who received either single or multiple doses of BAN2401. No participants experienced TEAEs that led to discontinuation or death. The incidence of ARIA‐E/H on MRI was similar between those who received BAN2401 and those on a placebo.

## 4 DISCUSSION

### 4.1 Summary of findings

The primary objective, a composite score, did not demonstrate a meaningful difference between the two groups in a Bayesian analysis after 12 months in a Phase 2b, dose‐finding study of 854 persons with early AD (Swanson et al., 2018). However, after 18 months, analyses revealed that lecanemab was able to clear amyloid in a dose‐ and time‐dependent manner, and the drug was associated with less clinical decline on some measures compared to the placebo (Swanson et al., 2018). The appropriate dose identified in the trial was 10 mg of lecanemab per kilogram of body weight (ED50), which was administered intravenously every 2 weeks, with a low incidence (9.9%) of ARIA, and less than 3% of those with ARIA experienced symptoms such as edema or effusions (ARIA) (Swanson et al., 2018). This was supported by the phase 3 trial, which shows significant clinical decline and reduction in amyloid burden but was associated with adverse events. Longer trials are warranted to determine the efficacy and safety of lecanemab in early AD (Ouzzani et al., 2016).

Logovinsky's study reported safety outcomes using staggered parallel single and MAD (Wisniewski & Goñi, 2015). The most common adverse effect was dizziness, fatigue, sinusitis, upper respiratory tract infection, headache, and orthostatic hypotension (Logovinsky et al., 2016). No symptomatic ARIA‐Es, ARIA‐Es, or superficial hemosiderosis were observed in participants who received either single or multiple doses of BAN2401, and the incidence of ARIA‐E/H on MRI was similar between those who received BAN2401 and those on placebo (Logovinsky et al., 2016).

### 4.2 Agreement and disagreement with contemporary research

Lecanemab is a new drug, and research is still going on to find out the efficacy and safety of this drug. So there is very limited data supporting its efficacy and adverse effects. FDA has approved this drug through an accelerated pathway based on its Phase 2 clinical trial data (Food and Drug Administration (FDA) (2023); Rodriquez, 2023). Based on the disappearance of amyloid plaques observed on PET scans, the FDA approved anti‐amyloid antibodies (Verger et al., 2023). But the majority of the cleared amyloid was located in the plaque‐free white matter. This raises doubts about the approval procedure (Høilund‐Carlsen et al., 2022). Because amyloid plaques are tiny and distributed in size, PET scans have difficulty imaging them, especially in the early stages of the disease (Alavi et al., 2020). AD pathology primarily affects the temporoparietal lobes; however, amyloid imaging frequently reveals uptake in the frontal lobes (Marcus et al., 2014; Sintini et al., 2019). Even though it shows more promising results than most other monoclonal antibodies there is some concern about the cost of the drug (Selkoe, 2019; 2023). Moreover, there are differences in response found in the subpopulation of the groups (Ouzzani et al., 2016). Takeshi Iwatsubo raised a few important questions regarding the challenges of using lecanemab that need to be found out (Iwatsubo, 2023) So further extensive research is recommended by them.

### 4.3 Research implications

Lecanemab shows promising results in early AD (Ouzzani et al., 2016; Swanson et al., 2018). But there are some important adverse effects that can happen along with its use (Swanson et al., 2021). So cautious measures should be taken before the use of lecanemab. Patients with ARIAs have severe damage to their white and gray matter. Those who receive these therapies frequently experience these anomalies, which point to permanent brain injury (Ouzzani et al., 2016; Swanson et al., 2018). The removal of amyloid plaques observed on PET scans of Alzheimer's patients may be explained by the damage. To evaluate the deleterious consequences, FDG‐PET imaging should be used in further research both before and after antibody therapy (Høilund‐Carlsen et al., 2023). Additionally, new research indicates that antibody therapies significantly reduce the amount of brain tissue as determined by MRI (Høilund‐Carlsen et al., 2023).

This research also finds out that there are still many questions that are not been answered yet (Ouzzani et al., 2016). So further extensive research is needed to find out those answers, which might open a new door for the treatment of AD.

### 4.4 Strengths and limitations

We adhered closely to the PRISMA recommendations when conducting this systematic review. This analysis only included RCTs. The Cochrane ROB evaluation method was used to critically evaluate the included publications for bias risk.

There are obviously some drawbacks to this study. Only English‐language articles were taken into consideration. Therefore, it is possible that studies published in any other language may be overlooked. Second, we only included a small number of RCTs with adequate sample sizes. Thus, the outcomes might occasionally be inflated.

## 5 CONCLUSION

Although the Phase 3 trial's results are undoubtedly encouraging, there are still a lot of unanswered concerns regarding lecanemab and its ability to treat AD. One important question is whether the benefits seen in the Phase 3 trial will translate into improved clinical outcomes for patients (van Dyck et al., 2023). Another concern is whether lecanemab will be beneficial in people with more advanced types of AD when the pathology is more severe and extensive (Høilund‐Carlsen et al., 2022; Rodriquez, 2023).

The results of the Phase 3 study represent a substantial advancement in the creation of therapies for AD notwithstanding these concerns. Lecanemab is a novel strategy for addressing the pathology at the root of the illness, and the positive results of the Phase 3 study offer encouragement to patients and their families (Rodriquez, 2023). Lecanemab's safety and effectiveness still need to be further investigated, but the findings of the Phase 3 trial indicate that it might significantly advance AD therapy choices (Rodriquez, 2023).

There are other medications now being developed for the treatment of AD in addition to lecanemab. These include aducanumab, another A‐targeting monoclonal antibody, as well as a number of small‐molecule medications (Cummings et al., 2019; Verger et al., 2023). Although several of these medications showed promise in preclinical and early‐stage clinical studies, failure in late‐stage trials highlights the need for more research and development of fresh AD therapies.

## AUTHOR CONTRIBUTIONS

**Md Fahad Hossain**: Conception and design; methodology; database search; protocol writing; risk of bias; supervision; writing—original draft; writing—review and editing. **Ashma Ul Husna**: Inclusion and exclusion; writing—original draft; writing—review and editing. **Manish Kharel**: Data collection; data extraction; protocol writing; writing—original draft; writing—review and editing.

## CONFLICT OF INTEREST STATEMENT

The authors declare that they have no conflicts of interest.

### PEER REVIEW

The peer review history for this article is available at https://publons.com/publon/10.1002/brb3.3592.

## Data Availability

The deidentified data that support the findings of this review are presented in the text.
